# Clinical relevance of aortic conduit and reservoir function

**DOI:** 10.1136/openhrt-2024-002713

**Published:** 2024-08-19

**Authors:** Hosamadin Assadi, Chris Sawh, Hilmar Spohr, Faye Nelthorpe, Sunil Nair, Marina Hughes, David Ashman, Alisdair Ryding, Gareth Matthews, Rui Li, Ciaran Grafton-Clarke, Zia Mehmood, Abdallah Al-Mohammad, Bahman Kasmai, Vassilios S Vassiliou, Pankaj Garg

**Affiliations:** 1 Department of Cardiovascular and Metabolic Health, University of East Anglia, Norwich, UK; 2 Norfolk and Norwich University Hospitals NHS Foundation Trust, Norwich, UK; 3 Department of Infection, Immunity & Cardiovascular Medicine, The University of Sheffield, Sheffield, UK; 4 South Yorkshire Cardiothoracic Centre, Sheffield Teaching Hospitals NHS Foundation Trust, Sheffield, UK; 5 University of East Anglia, Norwich, UK

**Keywords:** heart failure, magnetic resonance imaging, aortic diseases

## Abstract

**Background:**

Aortic conduit and reservoir functions can be directly measured by four-dimensional flow (4D flow) cardiovascular magnetic resonance (CMR).

**Methods:**

Twenty healthy controls (10 young and 10 age-gender-matched old controls) and 20 patients with heart failure with preserved ejection fraction (HFpEF) were recruited. All had 4D flow CMR. Flow was quantified at the ascending and descending aorta levels. In addition, at the ascending aorta level, we quantified systolic flow displacement (FDs) and systolic flow reversal ratio (sFRR). The aortic conduit function was defined as the relative drop in systolic flow from the ascending to the descending aorta (∆Fs). Aortic reservoir function was defined as descending aortic diastolic stroke volume (DAo SV_d_).

**Results:**

Both ∆Fs (R=0.51, p=0.001) and DAo SV_d_ (R=−0.68, p=0.001) were significantly associated with ageing. Native T1 (R=0.51, p=0.001) and extracellular volume (R=0.51, p=0.001) showed maximum association with ∆Fs. ∆Fs significantly increased in HFpEF versus age-gender-matched controls (41±8% vs 52±12%, p=0.02). In multiple regression, only ∆Fs and DAo SV_d_ were independent predictors of the estimated glomerular filtration rate (model R=0.77, p=0.0001). FDs was significantly associated with ∆Fs (R=0.4, p=0.01) and DAo SV_d_ (R=−0.48, p=0.002), whereas sFRR was mainly associated with DAo SV_d_ (R=−0.46, p=0.003).

**Conclusion:**

Both aortic conduit and reservoir function decline with age and this decline in aortic function is also independently associated with renal functional decline. Ascending aortic turbulent flow signatures are associated with loss of aortic conduit and reservoir functions. Finally, in HFpEF, aortic conduit and reservoir function demonstrate progressive decline.

**Trials registration number:**

NCT05114785.

**WHAT IS ALREADY KNOWN ON THIS TOPIC**

- Direct non-invasive measurement of aortic conduit and reservoir functions remains an unmet clinical need.

**WHAT THIS STUDY ADDS**

- This study shows that four-dimensional flow cardiovascular magnetic resonance (CMR) can directly measure aortic conduit and reservoir functions.
- The decline of aortic conduit and reservoir function is associated with turbulence in the ascending aorta, ageing, renal impairment and symptoms.

**HOW THIS STUDY MIGHT AFFECT RESEARCH, PRACTICE OR POLICY**

- The findings introduce a new non-invasive method for assessing aortic functions, potentially improving cardiovascular risk management.
- Understanding aortic flow patterns’ impact on organ perfusion could guide future therapies and inform the clinical use of advanced CMR techniques.

## Introduction

 The aorta serves two primary functions: conduit and reservoir.^1^ The conduit function of the aorta refers to its role as a passage for blood ejected from the left ventricle during systole, delivering it to the systemic circulation. Conversely, the reservoir function of the aorta involves its capacity to store a portion of the stroke volume during systole and release it during diastole. This function is facilitated by the compliance and elasticity of the aorta—the Windkessel function. This function is akin to the behaviour of a Windkessel (German for ‘air chamber’) in hydraulic systems, which smooths out fluctuations in fluid flow. In the cardiovascular system, the aorta expands during systole to absorb some of the blood ejected by the left ventricle and then recoils during diastole to help maintain continuous blood flow through the circulation. This elasticity helps to reduce the workload on the heart and ensures that peripheral tissues receive a steady supply of blood, contributing to efficient cardiovascular function and reduced pulsatility of blood pressure. These dual functions are critical for attenuating the pulsatile nature of blood flow from the left ventricle, ensuring a more continuous and steady flow throughout the arterial system. They also contribute to reducing cardiac workload and maintaining efficient organ perfusion, particularly during diastole, such as in the coronary circulation.

To date, we have not developed non-invasive methods to directly measure the conduit and reservoir functions of the aorta. Surrogate markers of these functions, such as pulse wave velocity (PWV), have been extensively researched. The American College of Cardiology Foundation and the American Heart Association have acknowledged that while PWV provides predictive information for cardiovascular risk, technical issues currently limit its applicability primarily to research settings. These issues include the need for high temporal resolution, standardised measurement protocols, quality control procedures and the identification of risk-defining thresholds. Additionally, concerns about reproducibility and operator dependence limit the generalisability of findings derived from research studies.^2^

Cardiovascular magnetic resonance (CMR) imaging, specifically phase-contrast velocity-encoded imaging, is emerging as a reliable and reproducible method for quantifying aortic flow, thereby informing clinical decisions. Both two-dimensional phase contrast (2D PC) flow and four-dimensional (4D) flow CMR methods allow for the quantification of aortic flow and its complex patterns and signatures. A key strength of 4D flow CMR is its ability to directly measure aortic conduit and reservoir functions by quantifying proximal and distal aortic flows in a single acquisition.^3^ The proportion of blood flow from the proximal ascending aorta reaching the abdominal aorta can be used to directly quantify aortic conduit function, whereas the absolute measure of descending aortic distal diastolic forward flow is a direct measure of ascending aorta reservoir function.

Although it is plausible to postulate that these aortic functions will be associated with previously described flow abnormalities in the ascending aorta, such as flow displacement in systole (FDs) and systolic flow reversal ratio (sFRR), this has not been previously demonstrated.^4 5^ It also remains unclear how aortic conduit/reservoir function changes with ageing. Renal function declines with ageing, but it is unknown whether this decline is associated with aortic conduit or reservoir function. A recent study from our group shows that patients with heart failure with preserved ejection fraction (HFpEF), who predominantly exhibit advanced ageing and associated cardiovascular risk factors, have distinctly elevated FDs and sFRR.^6^ However, in this HFpEF population, it remains unknown whether the flow in the ascending aorta impacts aortic conduit or reservoir function.

Therefore, in this study, we hypothesise that 4D flow CMR can directly measure aortic conduit and reservoir functions, which are impacted by turbulence in the ascending aorta. We also postulate that aortic conduit and reservoir function are associated with ageing, renal impairment and symptom burden as measured by the New York Heart Association (NYHA) functional classification.

## Methods

### Study cohort

The study design was case-controlled cross-sectional observation study. We identified patients from the PREFER-CMR registry (ClinicalTrials.gov: NCT05114785) in Norfolk and Norwich University Hospitals. The PREFER-CMR registry is a prospective registry which recruits patients who have clinical CMR examination.

We identified 20 patients with HFpEF from the PREFER-CMR registry. The main inclusion criteria for patients were over 18 years of age and a confirmed clinical diagnosis of HFpEF. A confirmed clinical diagnosis of HFpEF needed to meet the following critieria as set in the European Society of Cardiology Guidelines and the UK HFpEF registry^7 8^: symptoms and signs of heart failure (HF), left ventricular ejection fraction (LVEF) ≥50% and objective evidence of cardiac structural and/or functional abnormalities consistent with the presence of LV diastolic dysfunction or raised LV filling pressures. The exclusion criteria were CMR contraindication or presence of any of the following: infiltrative cardiomyopathy, active myocarditis, constrictive pericarditis or cardiac tamponade, hypertrophic cardiomyopathy, arrhythmogenic right ventricular (RV) cardiomyopathy, severe primary valvular heart disease, idiopathic, heritable or drug-induced pulmonary arterial hypertension, heart transplantation or ventricular assist device and complex congenital heart disease.

We enrolled 20 healthy controls from the same registry. The principal inclusion criteria for the control group encompassed individuals who were aged >18 years, displayed no discernible manifestations of overt cardiovascular disease as determined by CMR and had a clinical rationale for undergoing CMR evaluation—mainly to rule out cardiovascular disease. Conversely, the exclusion criteria comprised obesity (body mass index >30 kg/m^2^), diagnosis of HF, compromised systolic function (defined as an LVEF <50%), the presence of myocardial scar or fibrosis, aortic regurgitation (AR) and elevated native T1 values exceeding 1050 ms using our bespoke normal range data. For a subsection of controls (50%), we recruited them by age-gender matching to the identified HFpEF cohort—this cohort was labelled as age-gender-matched old controls. The remaining 10, with lower ages than the HFpEF cohort, were labelled as the young cohort.

### STROBE statement

This study was conducted as per the STrengthening the Reporting of OBservational studies in Epidemiology statement and full checklist is included in the online supplemental file.

### Cardiac magnetic resonance protocol

CMR study was performed on a 1.5 T Magnetom Sola Siemens system with a superconducting magnet (Siemens Healthineers, Erlangen, Germany). All patients were examined in the supine position, headfirst, using a respiratory sensor and ECG gating. Additionally, the scanner was equipped with an 18-channel biometric body coil and a built-in 32-channel spine coil. All patients were cannulated with a wide-bore intravenous line for the contrast agent.

The CMR protocol included baseline survey images and functional cines, native T1 maps, gadolinium enhancement imaging, postcontrast T1 maps and 4D flow acquisition methods previously described by our group.^9^,^13^ For standard functional cines, we used electrocardiographic gating breath-hold technique and acquired 30 phases throughout the cardiac cycle. Other cine acquisition parameters include repetition time (TR): 2.71, echo time (TE): 1.13, field of view (FOV): 360×289.3 mm^2^ with phase FOV 80.4%, number of signal averages (NSA): 1, matrix: 224×180 (phase), bandwidth: 167.4 kHz (930 Hz/Px), flip angle: 80°, slice thickness: 8 mm and Grappa acceleration with a factor of 2.

Parametric mapping sequence for longitudinal (T1) relaxation time measurement was done using the Modified Look-Locker inversion recovery sequence. Sequence specifics include the following: TR: 2.64, TE: 1.09, FOV: 380×323.6 (phase FOV 85.2%) mm^2^, NSA: 1, matrix: 256×144 (phase), bandwidth: 156.24 kHz (1085 Hz/Px), flip angle: 35°, slice thickness: 8 and Grappa acceleration with a factor of 2. Pixel-wise maps (MyoMaps, Siemens Healthineers) were generated on the scanner using Siemens’ HeartFreeze Inline Motion Correction technology.

Post surveys, cines and native T1 mapping, 0.1 mmol/kg of a gadolinium contrast agent (gadobutrol—Gadovist, Bayer Shering Pharma, Berlin, Germany) was administered and flushed with 30 mL of isotonic saline. Immediately postcontrast delivery, late gadolinium enhancement images in three long-axis and a stack of short‐axis imaging planes were obtained with a breath‐hold phase‐sensitive inversion recovery sequence 10 min after the contrast injection. The inversion time was adjusted to null normal myocardium (typically between 250 and 350 ms as assessed by a TI‐scout acquisition). This was followed by a postcontrast T1‐mapping acquisition 15 min after the contrast injection in the same orientations as the precontrast T1 mapping.

For 4D flow acquisition, the initial maximum encoded velocity (VENC) setting was 150–200 cm/s for all healthy controls and HFpEF cases. For 4D flow, we acquired 30 phases throughout the cardiac cycle to keep the data consistent with cines and hence the acquired temporal resolution was approximately 40 ms but dependent on the heart rate. Other 4D flow acquisition parameters include TR: 4.98, TE: 2.71, FOV: 200×256.3 mm^2^, NSA: 1, acquired voxel size=3×3×3 mm^3^, bandwidth: 31.616 kHz (494 Hz/Px), flip angle: 5 and Grappa acceleration in the phase-encoding direction with a factor of 2 and slice direction of 1. The ECG was retrospectively gated with free breathing to avoid diastolic temporal blurring.

### CMR analysis

All volumetric assessments were done using CVI42 V.5.14 (Circle Cardiovascular Imaging, Calgary, Canada) in routine clinical workflow in the 4D lab (https://www.norwich4dlab.com). End-diastolic and end-systolic phases were manually defined, and contours were drawn automatically using artificial intelligence and then visually checked by an experienced operator. An experienced clinician with level 3 accreditation finally checked all postprocessing.

**Figure 1:**
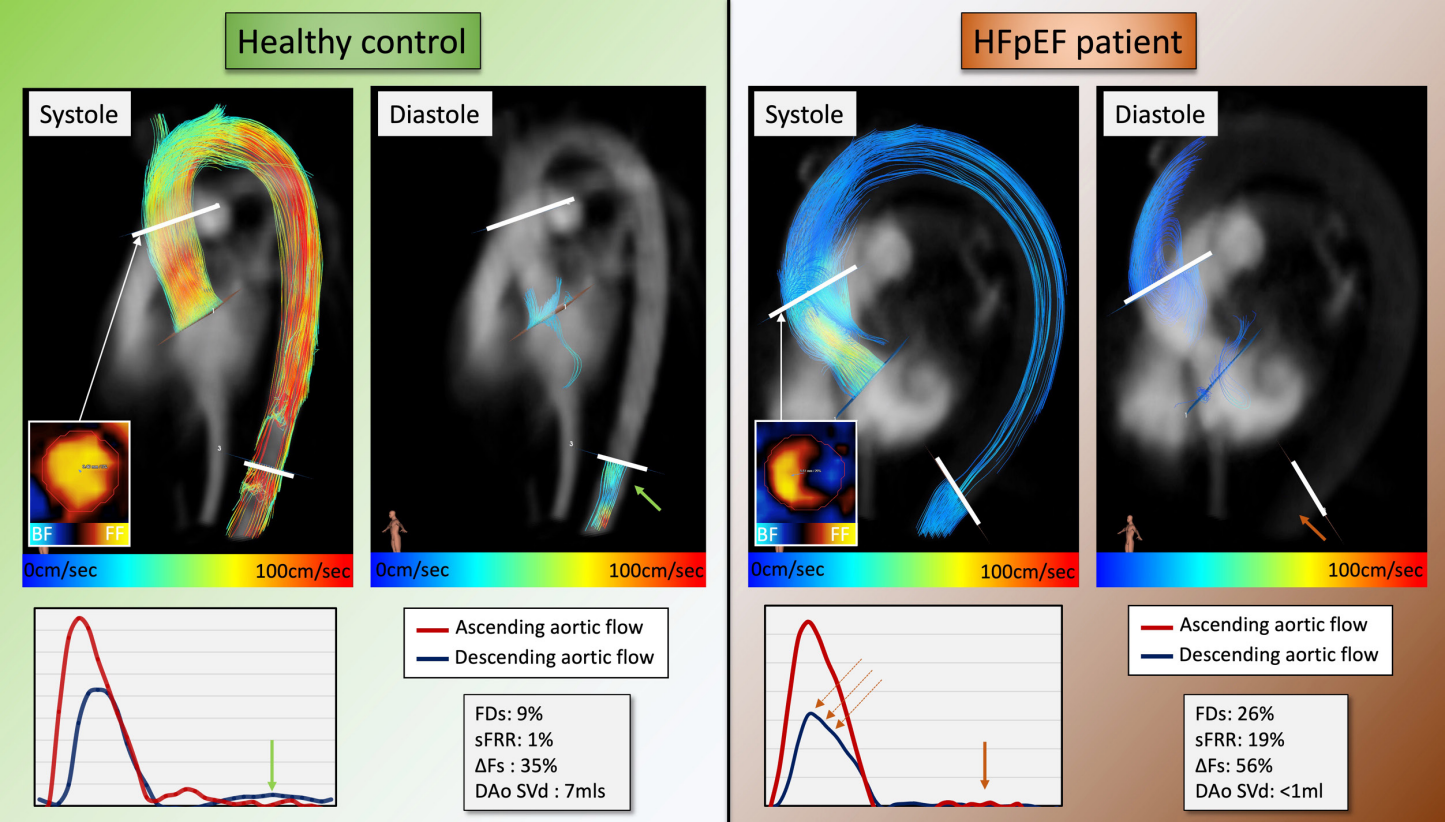
Two case examples from the study. (**A and B**) | Aortic flow streamlines in a healthy control. The velocity streamlines reach the distal aorta below the diaphragm with only moderate flow decrement (∆Fs only 35%). (**C and D**) | Aortic flow streamlines in a patient with heart failure with preserved ejection fraction (HFpEF). The ∆Fs increased to 56%, meaning descending aortic systolic flow, or aortic conduit function, was compromised. There was almost no flow during diastole, consistent with significantly impaired reservoir function. BF, backward flow; ∆Fs, systolic flow drop between ascending and descending aorta; DAo SV_d_, descending aortic diastolic stroke volume; FDs, flow displacement during systole; FF, forward flow; sFRR, systolic flow reversal ratio.

All image analyses were postprocessed with MASS research software (MASS, V.2023-EXP, Leiden University Medical Center, Leiden, The Netherlands). All CMR analyses were blinded to study demographics. A static reformatted plane was planned through the ascending aorta at the mid-main pulmonary artery level to generate a through-plane velocity encoded 2D PC data using 4D flow CMR data. This plane was treated as a 2D PC plane. Ascending aortic helical flow was defined as the flow swirling around the aortic centre line. Ascending aorta vortex flow was defined as any flow rotating on the long axis of the aorta near the inner curvature of the aortic root^14^ (figure 1).

The parameters delineated below were extrapolated from the aortic contours on the phase-contrast velocity-encoded reformatted plane:

1. Aortic forward flow refers to the stroke volume during a cardiac cycle.
2. Aortic backward flow: this encompasses the flow due to AR.
3. Ascending aortic maximum (Ao_max_) and minimum (Ao_min_) areas represent the largest and smallest cross-sectional areas, respectively, calculated in the ascending aorta during a cardiac cycle.
4. FDs: this is the distance between the vessel’s central point and the centre-of-velocity of the forward flow, normalised to the vessel size during systole. It is presented as a percentage. The centre-of-velocity of the forward flow is computed as the mean location of pixels weighted by the velocity values within a defined aortic contour on a 2D PC image. We also measured the late systolic flow displacement, which was averaged between the peak systole and the end of systolic flow. Averaged diastolic flow displacement was measured from end-systole to end of diastole.
5. Systolic forward and retrograde flows: these are obtained using per-pixel information. All positive velocities within the region of interest during systole were used to derive systolic forward flow. In contrast, all negative velocities within the same region were used to derive systolic retrograde flow.
6. sFRR: this ratio was derived from systolic forward and retrograde flows and was converted into a percentage by multiplying by 100. We also investigated FRR for the whole cardiac cycle.
7. Aortic conduit function: this was assessed by mapping distal descending aortic flow in the 4D flow data—this usually coincided with below diaphragm descending aorta level. The drop in forward flow during systole (∆Fs) at this level was derived using the following formula:
  [(Ascending aortic forward flow−descending aortic systolic forward flow)/ascending aortic forward flow]×100.
8. Aortic reservoir function: diastolic flow or stroke volume at the descending aortic level (DAo SV_d_) was directly measured to assess the aortic reservoir function.
  To avoid CMR acquisition-related noise in the aortic flow curve’s early and late diastolic phase, we only included the mid-diastolic flow rate at the 25th phase (S25). S25 was entered in the regression analysis to investigate the diagnostic power to differentiate age-gender-matched controls from patients with HFpEF.

### Statistical analysis

Data analyses were performed using MedCalc Statistical Software, V.22.014 (MedCalc Software, Ostend, Belgium), and OriginPro, V.2023 (OriginLab, Northampton, Massachusetts, USA). Continuous variables are presented as the mean and SD. All data were treated as parametric. To compare variables between the two groups, we employed an independent samples t-test. To investigate the association of all aortic flow indices to aortic conduit and reservoir function, we used Pearson’s product-moment correlation coefficient (R). In cases where a non-linear correlation was observed on scatter plot visualisation, we explored non-linear relationships using least-squares regression from curve fitting. For the time-resolved comparison of descending aortic flow, we used an independent samples t-test for each of the 30 phases of the cardiac cycle. For visual assessment of the p value, we generated 1−p-value curves to demonstrate the significance of graphic visualisation solutions better. To evaluate the ability of these blood flow parameters to distinguish HFpEF, we conducted a receiver operator characteristic analysis and used the Youden Index to determine cut-off values. Discrete data are presented as number (n) and percentage (%) with hypothesis testing using Fisher’s exact test. We deemed statistical significance at a threshold of p<0.05.

## Results

### Demographics and CMR characteristics

Patient demographics are summarised in table 1. In total, we recruited 20 controls and 20 patients with HFpEF. Compared with controls, individuals with HFpEF were significantly older (70±10 years vs 52±21 years, p=0.001) and had higher body mass index (25±4 kg/m^2^ vs 31±6 kg/m^2^, p=0.002). In addition, patients with HFpEF had a lower mean estimated glomerular filtration rate (eGFR) than controls (64±18 mL/min/1.73 m^2^ vs 87±7 mL/min/1.73m^2^, p<0.001).

**Table 1:** Study demographics

Variables	Controls (n=20)	HFpEF (n=20)	P value
Age, years	52±21	70±10	0.001
Female, n (%)	12 (60)	13 (65)	0.752
Body mass index, kg/m^2^	25±4	31±6	0.002
NYHA class >1, n (%)	3 (15)	14 (70)	<0.001
Diabetes mellitus, n (%)	1 (5)	5 (25)	0.08
Hypertension, n (%)	6 (30)	17 (85)	0.001
Cerebrovascular accidents, n (%)	0 (0)	3 (15)	0.075
Atrial fibrillation, n (%)	0 (0)	7 (35)	0.004
Hypercholesterolaemia, n (%)	6 (30)	12 (60)	0.059
Haemoglobin, g/L	143±11	137±15	0.19
Creatinine, μmol/L	72±14	96±27	0.002
eGFR, mL/min/1.73 m^2^	87±7	64±18	<0.001

eGFR, estimated glomerular filtration rate; HFpEF, heart failure with preserved ejection fraction; NYHA, New York Heart Association

Left heart and right heart CMR characteristics are summarised in table 2. While LVEF was significantly lower in the HFpEF group (58% vs 63%, p=0.029), left ventricular stroke volume was similar in both cohorts (p=0.943). No significant differences were observed in LV end-systolic volume (ESV) and end-diastolic volume (EDV) between the two groups (both p>0.05). Moreover, RV EDV, RV ESV, RV SV and RV EF were similar in both groups (p=0.78, p=0.77, p=0.84, p=0.58), respectively. Elevated native T1 values (p=0.04) and extracellular volume (ECV) (p=0.03) in the HFpEF group pointed towards potential myocardial fibrosis.

**Table 2:** Routine cardiovascular magnetic resonance imaging characteristics

Variables	Controls (n=20)	HFpEF (n=20)	P value
Left heart			
Left ventricular end-diastolic volume, mL	142±28	156±43	0.240
Left ventricular end-systolic volume, mL	52±14	67±29	0.054
Left ventricular stroke volume, mL	89±20	89±23	0.943
Left ventricular ejection fraction, %	63±6	58±9	0.029
Left ventricular mass, g	125±63	132±42	0.677
Native T1, ms	1003±31	1074±133	0.042
Extracellular volume, %	23±3	27±5	0.034
Right heart			
Right ventricular end-diastolic volume, mL	152±32	156±43	0.781
Right ventricular end-systolic volume, mL	66±19	68±28	0.765
Right ventricular stroke volume, mL	86±19	87±25	0.840
Right ventricular ejection fraction, %	58±6	56±9	0.577

The data isare presented as mean±standard deviationSD.

### Ascending aortic flow characteristics

**Figure 2:**
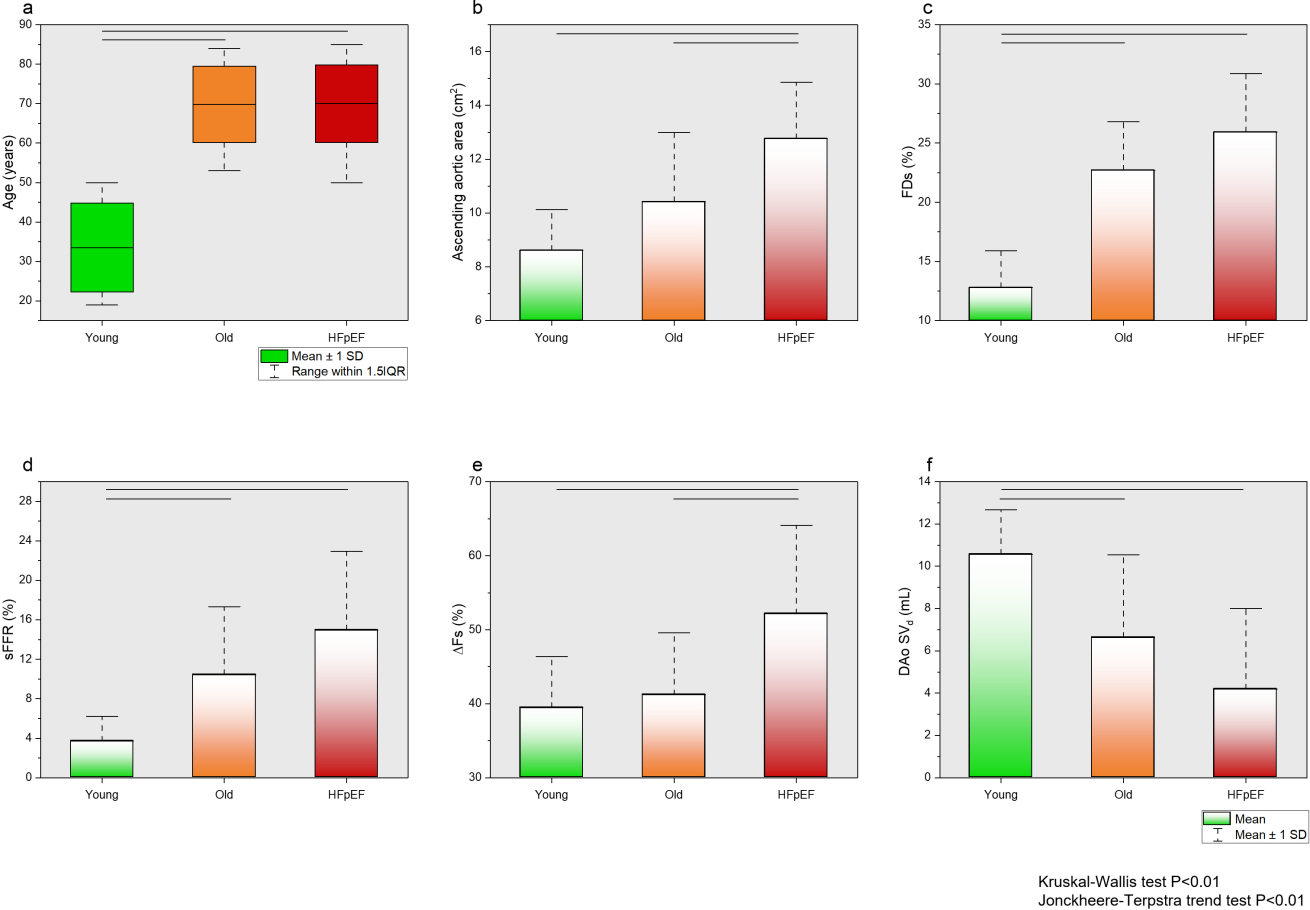
(**A**) | Box-whisker plot of age demonstrating comparable age between young and old and heart failure with preserved ejection fraction (HFpEF). (**B–F**) | Histogram plot of all aortic geometric and flow indices demonstrating differences in different ages and conditions. Ascending aortic area and ∆Fs significantly differ in older controls and patients with HFpEF. Nevertheless, the trend for change remains significant across all aortic flow metrics. ∆Fs, systolic flow drop between ascending and descending aorta; DAo SV_d_, descending aortic diastolic stroke volume; FDs, flow displacement during systole; sFRR, systolic flow reversal ratio.

The maximum cross-sectional area of the aorta (Ao_max_) and the minimum cross-sectional area of the aorta (Ao_min_) was significantly larger in patients with HFpEF compared with controls (13±2 cm² vs 10±2 cm² and 7±2 cm² vs 10±2 cm², both p<0.001) (table 3). The percentage of FDs and sFRR were significantly higher in the HFpEF group (p=0.001 for both). The systolic flow drop (∆Fs) was significantly higher in the HFpEF cohort (52±12%) compared with the control group (40±7%, p=0.001). Similarly, the diastolic stroke volume at the descending aortic level (DAo SV_d_ (mL)) was significantly lower in the HFpEF group (4±4 mL) compared with controls (9±4 mL, p=0.001) (figure 1—case examples). Histogram plots of all aortic geometric and flow indices demonstrating differences in different ages and conditions are illustrated in figure 2. The trend for change remains significant across all aortic flow metrics. However, ∆Fs significantly increased in age-gender-matched controls and patients with HFpEF.

**Table 3:** Ascending and descending aortic flow haemodynamics

	Controls (n=20)	HFpEF (n=20)	P value
Ascending aorta flow			
Ao_max_, cm^2^	10±2	13±2	<0.001
Ao_min_, cm^2^	7±2	10±2	<0.001
Heart rate, bpm	70±12	65±12	0.214
Net aortic forward flow, mL	74±15	77±16	0.680
Net aortic backward flow, mL	2±2	3±2	0.065
FDs, %	18±6	26±5	<0.001
Systolic forward flow, mL	76±17	86±17	0.065
Systolic retrograde flow, mL	5±6	13±8	0.001
sFRR, %	7±6	15±8	0.001
Descending aortic flow			
Systolic forward flow, mL	44±11	37±12	0.049
sFRR, %	1±1	2±2	0.180
Conduit and reservoir function			
∆Fs, %	40±7	52±12	0.001
DAo SV_d_, mL	9±4	4±4	0.001

The data isare presented as mean±standard deviationSD.

Ao_max_, maximum ascending aortic area; Ao_min_, minimum ascending aortic area; FDs, flow displacement during systole; ∆Fs, systolic flow drop between ascending and descending aorta; HFpEF, heart failure with preserved ejection fraction; sFRR, systolic flow reversal ratioSV_d_, descending aortic diastolic stroke volume

### Aortic conduit and reservoir function decline with age

**Figure 3:**
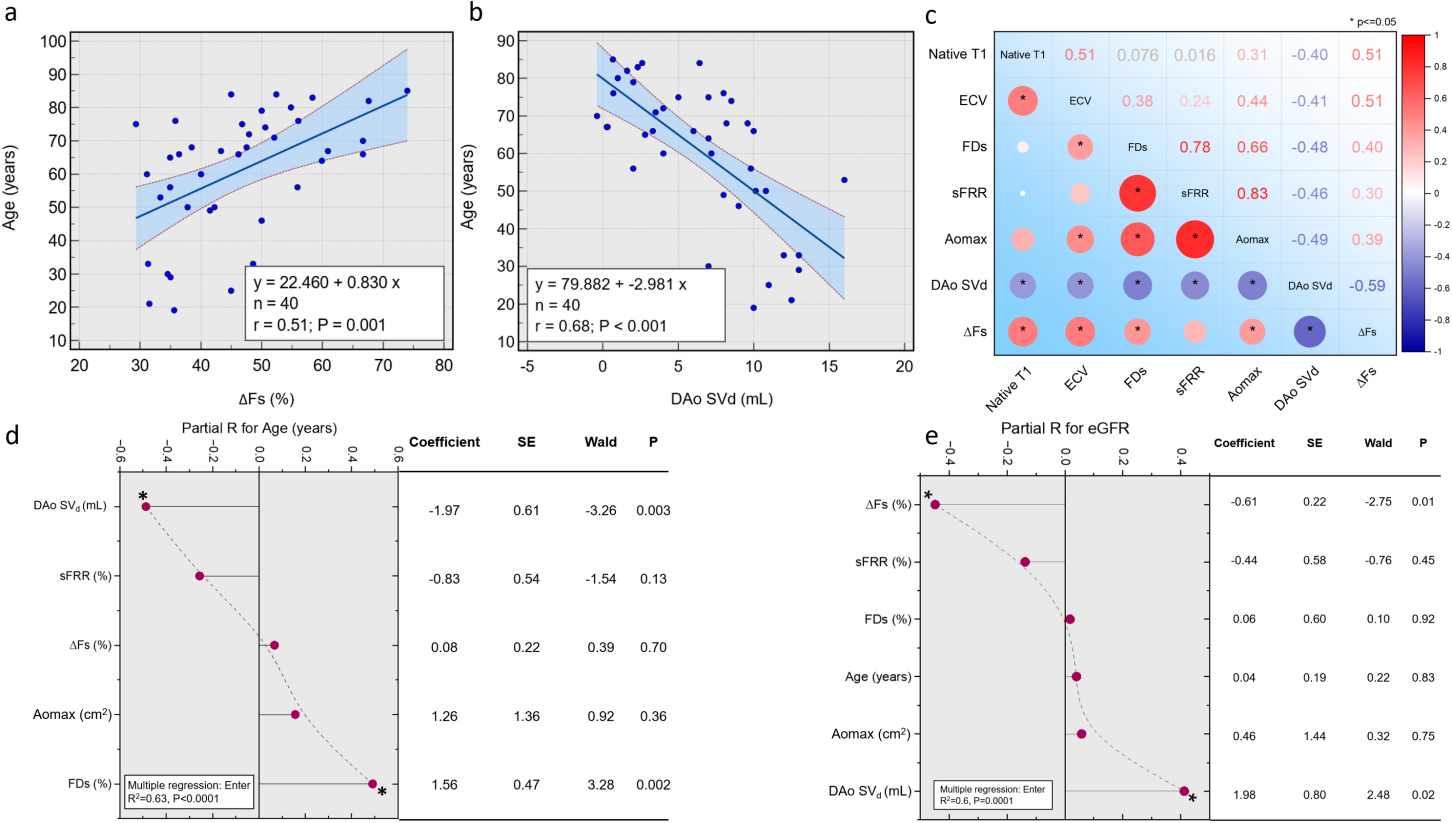
(**A and B**) | Scatter plots demonstrating association of age to aortic conduit and reservoir function. (**C**) | Correlation plot of myocardial tissue characterisation by native T1 and extracellular volume (ECV) to relevant aortic flow indices and metrics. We noted that native T1 and ECV were most associated with aortic conduit function measured by ∆Fs. (**D and E**) | Multiple regression results for age and estimated glomerular filtration rate (eGFR) association with aortic indices. Aortic reservoir function is independently associated with ageing. In addition, both aortic conduit and reservoir functions demonstrate a strong association with eGFR. ∆Fs, systolic flow drop between ascending and descending aorta; Ao_max_, maximum ascending aortic area; DAo SV_d_, descending aortic diastolic stroke volume; FDs, flow displacement during systole; sFRR, systolic flow reversal ratio.

We observed that both aortic conduit and reservoir function decline with the increasing age of our cohort. The association was more significant for the reservoir function (R=−0.68, p<0.001) than for the conduit function (R=0.51, p=0.001) (figure 3A,B). In multivariate regression of all relevant aortic parameters, aortic reservoir function was independently associated with ageing (figure 3D).

### Age-gender-matched controls versus patients with HFpEF

A detailed description of age-gender-matched controls versus HFpEF results is illustrated in online supplemental table S1. The groups were matched for age and gender. The HFpEF group had significantly higher weight (89±20 kg vs 71±13 kg, p=0.02), creatinine (96±27 µmol/L vs 70±15 µmol/L, p=0.02), eGFR (64±18 mL/min/1.73 m^2^ vs 83±9 mL/min/1.73 m^2^, p=0.01), LV ESV (67±29 mL vs 45±9 mL, p=0.03), Ao_max_ (13±2 cm^2^ vs 10±2 cm^2^, p=0.01), Ao_min_ (10±2 cm^2^ vs 8±2 cm^2^, p=0.01), ascending aortic systolic forward flow (86±17 mL vs 71±17 mL, p=0.03) and ΔFs (52±12% vs 41±8%, p=0.02) than age-gender-matched controls. These results suggest that patients with HFpEF have altered aortic haemodynamics compared with age-gender-matched controls.

### Association of ascending aortic flow haemodynamics with conduit and reservoir functions

We observed a significant correlation between the three phenotypic categories (young control, age-gender-matched controls and HFpEF cohort) and both aortic conduit ∆Fs and reservoir DAo SV_d_ functions with Pearson’s R values of 0.49 (p=0.001) and –0.61 (p<0.001), respectively (table 4). As the aortic area (Ao_max_) increased, aortic conduit function decreased with rise in ∆Fs and and also aortic reservoir function (DAo SV_d_) decreased. FDs (%) and sFRR (%) also showed significant correlations with reservoir function (R=–0.48 and –0.46, both p<0.01, respectively), and only FDs correlated significantly with the conduit function (R=0.40, p=0.01) (table 4). However, net aortic forward flow and systolic forward flow did not correlate significantly with either conduit or reservoir. Net aortic backward flow showed no significant correlation with ∆Fs but a significant negative correlation with DAo SV_d_ (Pearson’s R=–0.35, p=0.02) (table 4).

**Table 4:** Correlation between ascending aortic haemodynamics and the conduit function of the ascending aorta

Aortic flow parameters	Conduit (∆Fs (%))*		Reservoir (DAo SV_d_ (mL))†	
	R	P value	R	P value
Phenotypes (young, age-gender-matched, HFpEF)	0.49	0.001	–0.61	0.0001
Ao_max_, cm^2^	0.39	0.01	–0.49	0.001
Net aortic forward flow, mL	0.07	0.65	0.21	0.19
Net aortic backward flow, mL	–0.22	0.18	0.17	0.30
Systolic forward flow, mL	0.11	0.50	0.08	0.62
Systolic backward flow, mL	0.28	0.08	–0.35	0.02
FDs, %	0.40	0.01	–0.48	0.002
sFRR, %	0.30	0.06	–0.46	0.003
Native T1, ms	0.51	0.001	−0.40	0.02
Extracellular volume, %	0.51	0.001	−0.41	0.01

* Defined as a drop in flow from ascending aorta to descending aorta, ∆Fs.

† Defined as the diastolic stroke volume at the descending aortic level, DAo SV_d_.

Ao_max_, maximum ascending aortic area; FDs, flow displacement during systole; HFpEF, heart failure with preserved ejection fraction; sFRR, systolic flow reversal ratio

### Myocardial tissue changes link to aortic flow abnormalities

The results indicate a significant correlation between ∆Fs (%) and both native T1 (ms) and ECV (%), with correlation coefficients of 0.51 (p<0.01) for both (figure 3C). DAo SV_d_ (mL) also showed a significant negative correlation with both native T1 and ECV with correlation coefficients of –0.40 (p=0.02) and –0.41 (p=0.01), respectively (figure 3C). Ao_max_ (cm^2^) showed a moderate correlation with ECV (R=0.44, p=0.01), but the correlation with native T1 mapping was not significant (R=0.31, p=0.06). FDs (%) showed a moderate correlation with ECV (R=0.38, p=0.02), but the correlation with native T1 was not significant (R=0.08, p=0.66). sFRR (%) did not show any significant correlation with either native T1 or ECV.

### Renal function link to aortic flow abnormalities

The least squares multiple regression analysis (Enter method) was performed on the whole cohort (figure 3E). The coefficient of determination (R²) was 0.6, and the adjusted R² was 0.52 (p=0.0001). The multiple correlation coefficient was 0.77, and the residual SD was 12.46. The regression equation included several independent variables. ∆Fs (%) showed a significant negative correlation with eGFR (partial R=–0.45, p=0.01), and DAo SV_d_ (mL) showed a significant positive correlation (partial R=4.1, p=0.02). However, Ao_max_ (cm^2^), age (years), FDs (%) and sFRR (%) did not show any significant correlation with eGFR (all p>0.05). The analysis of variance showed a considerable F ratio of 7.46 (p=0.0001). The residuals were tested for normal distribution using the Shapiro-Wilk test, which accepted normality (W=0.96, p=0.28).

### Time-resolved descending aortic flow curves

**Figure 4:**
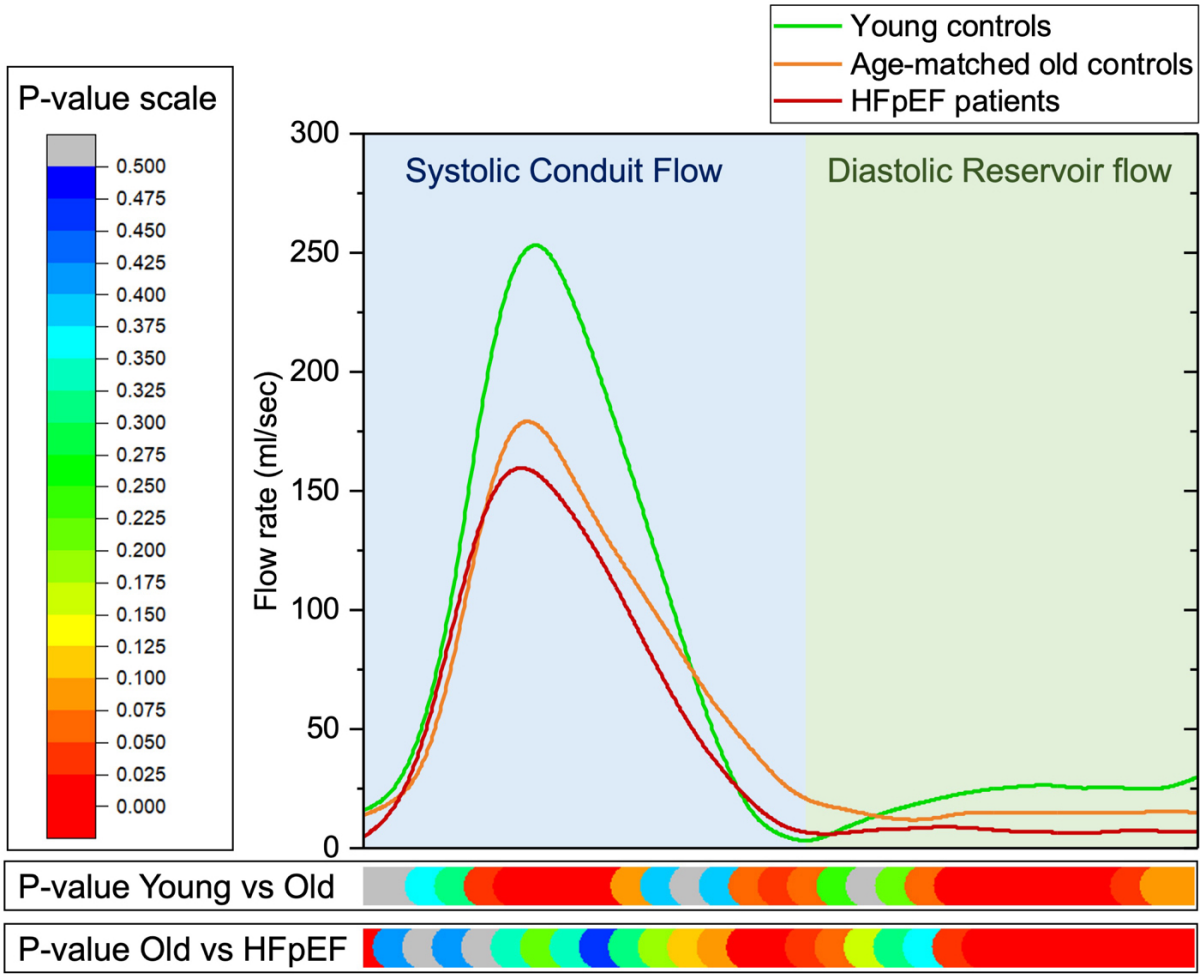
Averaged time-resolved descending aortic flow curves for young controls, old controls and patients with heart failure with preserved ejection fraction (HFpEF). There was a significant rise in mid-systolic flow in young controls versus older controls, and diastolic reservoir flow was significantly lower in older controls, too. In old controls versus patients with HFpEF, systolic conduit flow differences were insignificant, but significantly lower diastolic reservoir flow was observed.

We observed significant variations in time-resolved (30 cardiac phases) mean analysis of the descending aortic flow curves in age-gender-matched controls versus HFpEF (figure 4) (online supplemental table S2). The systolic descending aortic flow was significantly lower in patients with HFpEF than in age-gender-matched controls. Even though DAo SV_d_ (mL) was not statistically different between the age-gender-matched controls and HFpEF, we observed in time-resolved analysis that there were several phases in mid to late diastole where there was an obvious significant decline in flow rate in patients with HFpEF compared with age-gender-matched controls (figure 4) (online supplemental table S2).

### Aortic conduit and reservoir function link to symptom burden

We observed a significant difference in DAo SV_d_ between patients with stable NYHA functional class (<2) vs patients with high symptom burden (NYHA >2) (F ratio=5.9, p=0.02), with the higher NYHA class associated with a lower mean DAo SV_d_ (42±12 mL vs 29.7±7 mL, p=0.02). Similarly, we observed a trend with the higher NYHA class associated with higher ∆Fs (F ratio=3.5, 44±11% vs 54±11%, p=0.06).

## Discussion

This study is the first to describe and define aortic conduit and reservoir function clinically in a cohort of healthy controls and patients with HFpEF. The main findings of this study are that aortic conduit function, measured by the drop in systolic flow stroke volume from the ascending aorta to the descending abdominal aorta at the level just below the diaphragm, is significantly reduced in patients with HFpEF compared with age-matched controls. Both aortic conduit and reservoir function decline with age. Another important observation of this study is that ascending aortic flow turbulence, measured by FDs and sFRR are linked to aortic conduit and reservoir function. Finally, we observed that a decline in aortic conduit function is associated with a rise in myocardial fibrosis, measured by ECV and a decline in renal function is more strongly linked with aortic reservoir function.

Even though previous studies have assessed surrogate markers of aortic conduit and reservoir function using viscous dissipation or PWV,^15^ this remains the first study to measure the flow directly and define the aortic conduit and reservoir function in the adult population and patients with HFpEF.

### Aortic conduit function and its clinical relevance

The ∆Fs measured in this study can be easily derived either by 4D flow CMR covering the whole heart or a component below the diaphragm or by using two 2D PC CMR acquisitions—one at the ascending aortic level and one at the level of the descending aorta. This imaging biomarker reflects the systolic flow drop due to flow diversion to the upper body and due to flow abnormalities in the ascending aortic root. Higher distal pressure will also result in a lower pressure gradient between the ascending aorta and descending aorta, resulting in reduced flow in the descending aorta. We also observed that the ∆Fs was independently associated with FDs, an imaging biomarker of flow eccentricity. More recently, this imaging biomarker has been used as a therapeutic target in aortic valve intervention,^1 16^ and it also independently predicts aortic root dilatation.^17^ Flow eccentricity in the ascending aorta results in more turbulent flow and vortices forming near the inner curvature of the ascending aortic root. This results in energy dissipation during systolic phases and disrupts the laminar flow necessary for optimum conduit function. This phenomenon of increased ascending aortic flow eccentricity has been shown to have an independent detrimental effect on cardiac efficiency measured by VO_2_ max and reduction in exercise capacity in 169 healthy subjects.^5^

Moreover, our study builds on the existing literature and demonstrates that a rise in myocardial fibrosis measured by ECV is also associated with aortic conduit function. The aortic root remodels with advancing age, and the blood pressure rises. Both result in an increase in the afterloading conditions on the left ventricle, impacting the fibrotic burden. While this study is not intended for diagnostic assessment, it is important to highlight a noteworthy distinction between the healthy control group and patients with HFpEF. This observation underscores the pathophysiological sequelae associated with the disease, which has been previously described and possibly explains one of the multifactorial reasons for shortness of breath in HFpEF.

### Aortic reservoir function and its clinical relevance

In this study, we directly measured the distal diastolic descending aortic flow as an imaging biomarker of aortic reservoir function, also known as the Windkessel function, which is related to its elastic properties. Our assessment predominantly assesses the Windkessel function of the ascending aortic root, as our distal assessment is not further down in the descending aorta or its sub-branches. Our most striking observation was that in patients with HFpEF, there was hardly any diastolic descending aortic flow. Again, FDs independently predicted a reduction in aortic reservoir function. Furthermore, descending aortic diastolic flow was independently and most strongly associated with ageing, even when compared with aortic dimensions, which is one of the strongest predictors of ageing.^18^ We also noted that a decline in renal function (measured by the rise in serum creatinine) was independently linked with aortic reservoir function. Renal perfusion during diastole plays a crucial role in maintaining renal function and homeostasis. The kidneys receive approximately 20% of the cardiac output, and this blood flow is essential for glomerular filtration and tubular function. During diastole, blood flow to the kidneys continues due to the low resistance in the renal vasculature, which is critical for maintaining continuous perfusion and filtration.^19^ From the observations made in this study, it would be plausible to infer that renal perfusion depends more on the aortic Windkessel function than the conduit function. These findings could have significant implications, especially in developing mechanistic insight into cardiorenal syndrome. Furthermore, the aortic reservoir function appears to differentiate HFpEF from a healthy state better than the aortic conduit function. This makes sense as ageing, and creatinine were more associated with reservoir than conduit function, and they are markers of disease state in HFpEF.

### Limitations

This study has some limitations. One limitation of this study was that we did not recruit patients with aortic valve disease, namely aortic stenosis or bicuspid aortic valve. It is established that ascending aortic flow is abnormal in these study cohorts. Recent data suggest that flow abnormalities, namely FDs and FRR, are emerging as novel therapeutic targets in the aortic valve intervention.^16^ Future studies need to evaluate if a similar compromised aortic conduit and reservoir function pattern is noted in patients with aortic valve disease and linked with ascending aortic flow abnormalities. In addition, the assessment of the aortic reservoir function proposed in this work is less applicable to patients with significant aortic valve incompetency as they can have diastolic flow reversal even in a complaint aorta. Crucially, this study does not aim to provide a diagnostic evaluation. Rather, it is a mechanistic investigation that establishes the methodologies for direct functional assessment of the aortic conduit and reservoir, as opposed to relying on surrogate markers for these measurements. Hence, future studies need to evaluate the potential diagnostic advantage of directly assessing aortic conduit and reservoir function.

### Clinical implications

This is the first study to define the aortic conduit and reservoir function and demonstrate how the aortic function is associated with abnormal flow in the ascending aorta. Importantly, the mechanistic insights from our observations in this study reflect how aortic flow changes with ageing and how that impacts the aortic conduit and reservoir function. Furthermore, we show how the ascending aortic flow abnormalities are associated with the rise in native T1 values and ECV in the myocardium—illustrating myocardial-aortic physiological coupling. Of all the aortic flow and geometry indices, the aortic reservoir function, measured by DAo SV_d_, was independently associated with ageing. It would be plausible to conclude that ageing affects aortic reservoir function the most. A decrease in aortic reservoir function was associated with rising creatinine levels, confirming that a reduction in perfusion results in renal impairment. Future studies are warranted to explore what this means for patients, especially if they have gut-related symptoms due to reduced perfusion.

## Conclusion

This study defines the functional assessment of the aortic conduit and reservoir. Both aortic conduit and reservoir function decline with age, and this decline in aortic function is also associated with renal functional decline. The study further establishes a mechanistic association between ascending aortic turbulent flow signatures and the loss of aortic conduit and reservoir functions.

## supplementary material

10.1136/openhrt-2024-002713 online supplemental file 1

## Data Availability

Data are available on reasonable request.
