# Supplementary material for: Clinical relevance of aortic conduit and reservoir function
Source: Open Heart. 2024 Aug 19;11(2):e002713. doi: 10.1136/openhrt-2024-002713 (PMC11337677; doi:10.1136/openhrt-2024-002713)
Supplement: online supplemental file 1 [file openhrt-11-2-s001.docx]

**Supp Table S1**. Study demographics, cardiovascular magnetic resonance imaging characteristics, ascending and descending aortic flow haemodynamics for age-gender-matched controls, and heart failure with preserved ejection fraction (HFpEF) patients.

|  | *Age-gender-matched controls* | *HFpEF* cohort | *P* |
| --- | --- | --- | --- |
| Number | *10* | *20* | *-* |
| *Demographics* | | | |
| Age, years | 70±9 | 70±10 | 0.958 |
| Female, n (%) | 5 (50) | 13 (65) | 0.447 |
| Weight, kg | 71±13 | 89±20 | **0.019** |
| Height, cm | 168±6 | 170±8 | 0.469 |
| Body surface area, m^2^ | 1.9±0.33 | 2.01±0.25 | 0.333 |
| Diabetes mellitus, n (%) | 1 (10) | 5, (25) | 0.350 |
| Hypertension, n (%) | 5 (50) | 17, (85) | **0.042** |
| Myocardial infarction, n (%) | 1 (10) | 6, (30) | 0.236 |
| Cerebrovascular accidents, n (%) | 0 (0) | 3, (15) | 0.210 |
| Atrial fibrillation, n (%) | 0 (0) | 7, (35) | **0.033** |
| Hypercholesterolemia, n (%) | 4 (40) | 12, (60) | 0.317 |
| NT-proBNP, pg/mL | 206±144 | 856±891 | 0.179 |
| Haemoglobin, g/L | 141±14 | 137±15 | 0.535 |
| Creatinine, umol/L | 70±15 | 96±27 | **0.020** |
| eGFR, mL/min/1.73m^2^ | 83±9 | 64±18 | **0.014** |
| CMR characteristics | | | |
| Left ventricular end-diastolic volume, mL | 127±27 | 156±43 | 0.067 |
| Left ventricular end-systolic volume, mL | 45±9 | 67±29 | **0.030** |
| Left ventricular stroke volume, mL | 82±20 | 89±23 | 0.415 |
| Left ventricular ejection fraction, % | 64±4 | 58±9 | 0.050 |
| Left ventricular mass, g | 130±82 | 132±42 | 0.944 |
| Native T1, ms | 1000±39 | 1074±133 | 0.144 |
| Extracellular volume, % | 25±3 | 27±5 | 0.498 |
| Right ventricular end-diastolic volume, mL | 136±27 | 156±43 | 0.206 |
| Right ventricular end-systolic volume, mL | 61±19 | 68±28 | 0.474 |
| Right ventricular stroke volume, mL | 75±13 | 87±25 | 0.147 |
| Right ventricular ejection fraction, % | 56±7 | 56±9 | 0.830 |
| Ascending aortic flow haemodynamics | | | |
| Ao_max_, cm^2^ | 10±2 | 13±2 | **0.012** |
| Ao_min_, cm^2^ | 8±2 | 10±2 | **0.014** |
| Heart rate, bpm | 71±16 | 65±12 | 0.346 |
| Net aortic forward flow, mL | 65±13 | 77±16 | 0.057 |
| Net aortic backward flow, mL | 3±3 | 3±2 | 0.629 |
| FDs, % | 23±4 | 26±5 | 0.086 |
| Systolic forward flow, mL | 71±17 | 86±17 | **0.033** |
| Systolic retrograde flow, mL | 8±7 | 13±8 | 0.111 |
| sFRR, % | 10±7 | 15±8 | 0.141 |
| Descending aortic flow haemodynamics | | | |
|  |  |  |  |
| Systolic forward flow, mL | 38±8 | 37±12 | 0.759 |
| sFRR, % | 1±1 | 2±2 | 0.067 |
| Conduit and reservoir function | | | |
| ∆Fs, % | 41±8 | 52±12 | **0.016** |
| DAo SV_d_, mL | 7±4 | 4±4 | 0.112 |

*Abbreviations: Ao_max_,* *maximum ascending aortic area; Ao_min_, minimum ascending aortic area; DAo SV_d_, descending aortic diastolic stroke volume; eGFR, estimated glomerular filtration rate; ∆Fs, systolic flow drop between ascending and descending aorta; FDs,* *flow displacement during systole; HFpEF, heart failure with preserved ejection fraction; NT-proBNP, N-terminal pro-b-type natriuretic peptide; sFRR,* *systolic flow reversal ratio.*

**Supp Table S2 –** Time-resolved (30 cardiac phases) descending aortic flow curves mean ± standard deviation for age-gender-matched controls and heart failure with preserved ejection fraction (HFpEF) patients.

| **Variable** | | **Age-gender-matched controls** | **HFpEF** |  | | |
| --- | --- | --- | --- | --- | --- | --- |
|  |  | Mean±SD | Mean±SD | Bias | 95% CI | P ^a^ |
| Systole | S1 | 14.5±8 | 4.6±8 | –9.8 | –16.37 to –3.28 | 0.005 |
| Systole | S2 | 19.3±13 | 13.6±20 | –5.7 | –19.70 to 8.38 | 0.416 |
| Systole | S3 | 31.1±17 | 41.9±52 | 10.8 | –24.21 to 45.81 | 0.533 |
| Systole | S4 | 77.3±41 | 95.4±64 | 18.2 | –27.64 to 63.95 | 0.424 |
| Systole | S5 | 142.9±39 | 144.5±62 | 1.5 | –42.81 to 45.83 | 0.945 |
| Systole | S6 | 181.9±43 | 163.3±53 | –18.6 | –58.01 to 20.91 | 0.344 |
| Systole | S7 | 178.5±36 | 157.1±47 | –21.4 | –56.24 to 13.42 | 0.218 |
| Systole | S8 | 155.8±31 | 141.4±43 | –14.5 | –45.62 to 16.69 | 0.350 |
| Systole | S9 | 132.6±36 | 120.8±42 | –11.7 | –43.41 to 19.92 | 0.454 |
| Systole | S10 | 113.8±41 | 97.5±42 | –16.3 | –49.61 to 16.99 | 0.325 |
| Systole | S11 | 94.6±44 | 72.2±43 | –22.3 | –56.75 to 12.11 | 0.195 |
| Systole | S12 | 74.9±42 | 49.8±40 | –25.1 | –57.35 to 7.18 | 0.123 |
| Systole | S13 | 56.3±37 | 32.7±33 | –23.6 | –50.60 to 3.43 | 0.085 |
| Systole | S14 | 42.3±28 | 19.5±19 | –22.8 | –40.67 to –4.91 | 0.014 |
| Systole | S15 | 27.1±26 | 9.0±12 | –18.1 | –31.92 to –4.33 | 0.012 |
| Systole | S16 | 19.4±25 | 5.5±9 | –13.9 | –26.70 to –1.08 | 0.035 |
| Diastole | S17 | 16.6±19 | 6.4±10 | –10.1 | –20.75 to 0.52 | 0.062 |
| Diastole | S18 | 13.8±11 | 8.3±9 | –5.5 | –13.18 to 2.20 | 0.155 |
| Diastole | S19 | 12.0±9 | 8.2±10 | –3.8 | –11.61 to 3.96 | 0.324 |
| Diastole | S20 | 12.3±8 | 9.1±10 | –3.2 | –10.61 to 4.12 | 0.374 |
| Diastole | S21 | 14.9± | 9.2±7 | –5.7 | –11.17 to –0.15 | 0.044 |
| Diastole | S22 | 15.4±6 | 7.9±8 | –7.5 | –13.55 to –1.39 | 0.018 |
| Diastole | S23 | 15.2±5 | 7.4±8 | –7.8 | –13.67 to –1.85 | 0.012 |
| Diastole | S24 | 14.7±5 | 7.2±8 | –7.5 | –13.35 to –1.62 | 0.014 |
| Diastole | S25 | 14.8±5 | 6.0±10 | –8.7 | –15.41 to –2.05 | 0.012 |
| Diastole | S26 | 14.9±5 | 7.3±8 | –7.6 | –13.56 to –1.70 | 0.014 |
| Diastole | S27 | 15.2±7 | 7.6±8 | –7.5 | –13.53 to –1.54 | 0.016 |
| Diastole | S28 | 15.9±7 | 7.4±7 | –8.4 | –13.84 to –3.04 | 0.003 |
| Diastole | S29 | 15.4±7 | 7.1±7 | –8.4 | –13.85 to –2.91 | 0.004 |
| Diastole | S30 | 13.8±7 | 3.6±13 | –10.2 | –19.44 to –0.97 | 0.032 |

^a^ T-test.

**Study flow chart**

**Study Design: Case-controlled cross-sectional observation study.**

**Patient Identification**

- Source: PREFER-CMR registry (ClinicalTrials.gov: NCT05114785) in Norfolk and Norwich University Hospitals.

- The PREFER-CMR registry is a prospective registry which recruits patients who have clinical cardiac magnetic resonance imaging (CMR) examination.

Patient Groups

**HFpEF Patients**

- Identified **20 patients** with heart failure with preserved ejection fraction (HFpEF) from the PREFER-CMR registry.

- **Inclusion Criteria:** Over 18 years of age and a confirmed clinical diagnosis of HFpEF.

- **Exclusion Criteria:** CMR contraindication or presence of any of the following: infiltrative cardiomyopathy, active myocarditis, constrictive pericarditis or cardiac tamponade, hypertrophic cardiomyopathy, arrhythmogenic right ventricular cardiomyopathy, severe primary valvular heart disease, idiopathic, heritable or drug-induced pulmonary arterial hypertension, heart transplantation or ventricular assist device and complex congenital heart disease.

**Healthy Controls**

- Enrolled **20 healthy** **controls** from the same registry.

- **Inclusion Criteria:** Individuals who were aged more than 18 years, displayed no discernible manifestations of overt cardiovascular disease as determined by CMR, and had a clinical rationale for undergoing CMR evaluation – mainly to rule out cardiovascular disease.

- **Exclusion Criteria:** Obesity (body mass index >30kg/m2), diagnosis of HF, compromised systolic function (defined as a LV EF below 50%), the presence of myocardial scar or fibrosis, aortic regurgitation (AR) and elevated native T1 values exceeding 1050 milliseconds using our bespoke normal range data.

- For a subsection of controls (50%), we recruited them by **age-gender matching** to the identified HFpEF cohort.
